# Research on meat quality of Qianhua Mutton Merino sheep and Small-tail Han sheep

**DOI:** 10.1515/biol-2022-0493

**Published:** 2022-09-27

**Authors:** Limin Sun, Huaizhi Jiang

**Affiliations:** College of Animal Science and Technology, Jilin Agricultural University, Changchun, 130118, China

**Keywords:** Qianhua Mutton Merino, nutrient composition, amino acid, fatty acid, muscle fiber

## Abstract

In this study, we analyzed the meat quality of Qianhua Mutton Merino sheep (QHMM). QHMM and Small-tail Han sheep (STH) were selected as the test animals. After slaughtering, the longissimus dorsi muscles were tested to determine the nutrient composition, content of amino acid, fatty acid, and muscle fiber diameter. According to the nutrient test result, the protein content of QHMM was higher than that of STH. However, the fat content was significantly lower (*P* < 0.05) than STH. Additionally, total amino acid content, total essential amino acid content, total half-essential amino acid content, total non-essential amino acid content, and flavor amino acid content of QHMM were significantly higher (*P* < 0.05) than those of STH. Moreover, the contents of C22:1 n9, C18:2 n6, C18:3 n6, C22:6 n6, and C10:0 of the muscle in QHMM were significantly higher (*P* < 0.05) than those of STH. Furthermore, the essential amino acid score, the total unsaturated-to-saturated fatty acid ratio, and the polyunsaturated-to-saturated fatty acid ratio of QHMM were greater than those of STH. Additionally, the muscle fiber diameter of QHMM was considerably lower (*P* < 0.01) than that of STH. In conclusion, the meat quality of QHMM was better than that of STH.

## 1 Introduction

As an emerging sector in China’s animal husbandry industry, the mutton sheep sector plays a vital role in improving the agricultural production capability and people’s livelihood. Sheep are the primary source of mutton in China. With the gradual improvement in people’s living standards and change in diet, mutton has become a popular food due to its high protein, low fat, and low cholesterol levels. The demand for high-quality mutton has increased globally [1,2,3]. In 2021, the mutton output in China reached 5.14 million tons [4], which is the highest in the world. However, the sheep’s average carcass weight in China (only 16 kg) is still lower than that in countries with a highly developed mutton sheep sector (over 20 kg). Although there are more than 70 sheep breeds (Genetic resources) in China [5], only very few breeds with remarkable regionalization, for example, Bamei mutton sheep, Zhaoda mutton sheep, and Chahar mutton sheep, are specialized species subjected to systematic selection and with good meat production performance [6,7]. All other breeds are non-specialized local breeds and wool-mutton breeds.

**Figure 1:**
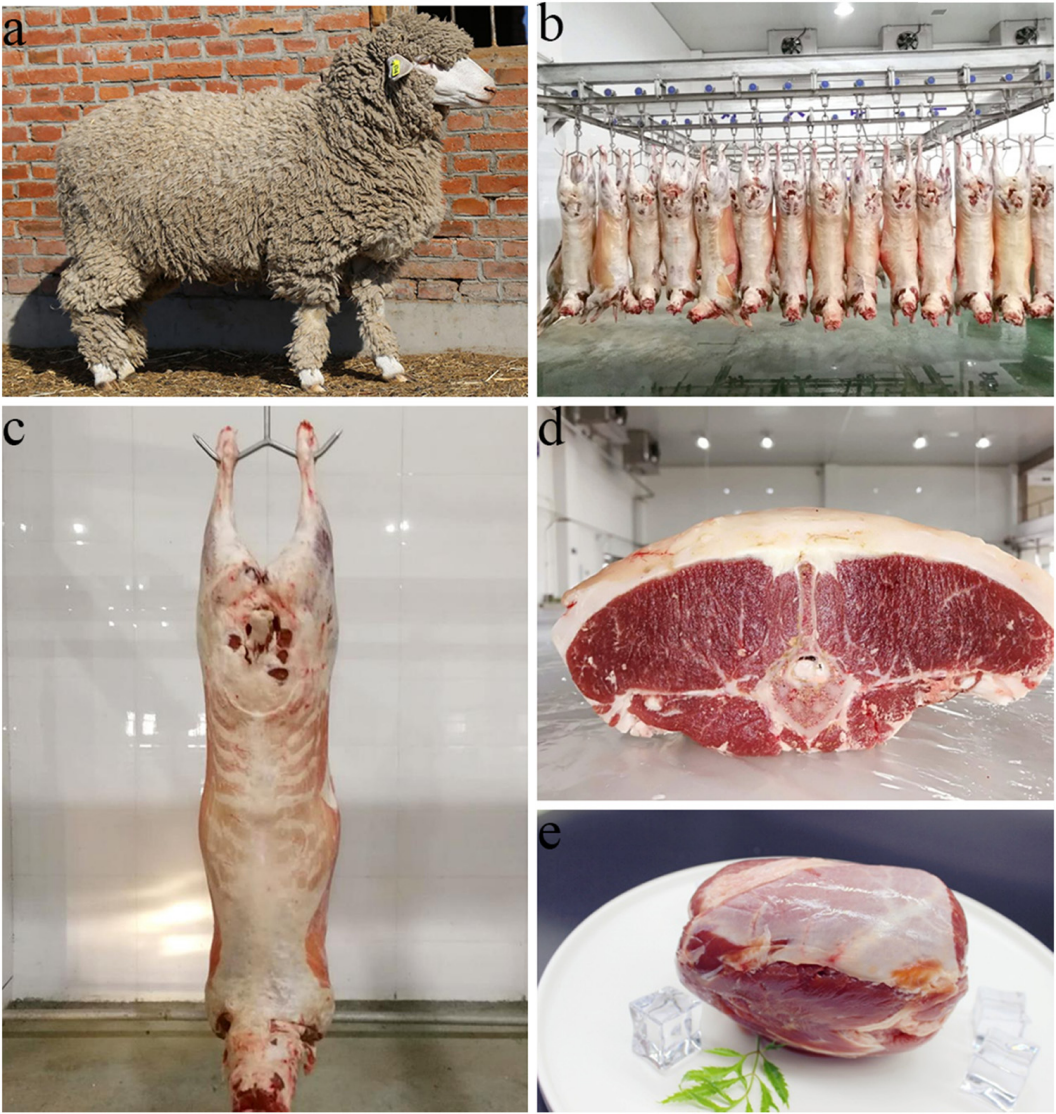
The body shape, appearance, and carcass characteristics of QHMM. (a) is the body shape, (b and c) are the carcass characteristics, (d and e) are the meat quality.

Qianhua Mutton Merino sheep (QHMM) is a new sheep breed with the characteristics of both meat and wool (as shown in Figure 1), which was bred in 2018 with the imported South African Mutton Merino Sheep as the male parent and the local Northeast Fine Wool Sheep containing the Australian Merino gene as the female parent, using the combination of conventional breeding methods and modern molecular breeding technology [8]. The breed not only has high meat production performance but also has excellent meat quality. It is the first meat Merino Sheep breed with independent intellectual property rights in China. Small-tail Han sheep (STH) is a female parent breed widely used in mutton sheep production in the Jilin Province and the northern area of China and is known for its high lambing rate and strong disease resistance [9,10,11]. However, its meat production is relatively low and the meat quality is not very high. No information was available on the nutrient composition and quality evaluation for QHMM and STH. The basic nutritional components (the content of moisture, protein, and fat), amino acid and fatty acid composition, and muscle fiber diameter are important indicators to evaluate the meat quality of sheep. Therefore, the analysis of the nutrient composition of the muscle tissue was performed in this study for QHMM and STH to reveal the differences between the mutton sheep breed currently cultivated and the local sheep breed widely used. A comprehensive comparison and evaluation of the nutrient composition of these two breeds provided a theoretical foundation for the cultivation of mutton sheep.

## 2 Materials and methods

### 2.1 Sample collection

This test was performed at Jilin Qian’an Zhihua Sheep Breeding Co., Ltd (44°44′25.3′′N and 124°0′11′′E) based on a randomized block design with 6 parallel in each group. In the test process, six female QHMM and six female STH yearlings, with similar initial weight and raised under the same conditions with free access to food (grass and concentrate) and water were selected as the test animals. The carcass trait including slaughter weight was reported in previous studies [12]. After the test animals were slaughtered, their longissimus dorsi muscles were collected and stored in a refrigerator at –20°C for subsequent meat quality testing. Their muscle samples, with an area of 1 cm^2^, were collected and fixed in 4% paraformaldehyde solution for subsequent meat slice preparation.


**Ethical approval:** The research related to animal use has been complied with all the relevant national regulations and institutional policies for the care and use of animals, and has been approved by the Laboratory Animal Welfare and Ethics Committee of Jilin Agricultural University (No. 2021 05 20 001).

### 2.2 Test methods

#### 2.2.1 Determining the basic nutrient composition

The muscle tissues of QHMM and STH were tested for determining the contents of protein, water, and fat following the methods described in GB/T 5009.5-2010 “*Determination of protein in foods*” [13], GB/T 5009.3-2010 “*Determination of moisture in foods*” [14], and GB/T 5009.6-2003 “*Determination of fat in foods*” [15], respectively.

#### 2.2.2 Determining the amino acid content

The muscle tissues of QHMM and STH were tested for determining the contents of 18 amino acids following the methods described in GB/T 5009.124-2003 “*Determination of amino acids in foods*” [16].

#### 2.2.3 Determining the fatty acid content

The muscle tissues of QHMM and STH were analyzed to determine the contents of fatty acids following the methods described in GB17376-17377-2008 “*Animal and vegetable fats and oils* – *Analysis by gas chromatography of methyl esters of fatty acids*” [17] and GB/T22223-2008 “*Determination of total fat, saturated fat, and unsaturated fat in foods* – *Hydrolytic extraction-gas chromatography*” [18].

#### 2.2.4 Determining muscle fiber diameter

The muscle samples fixed in 4% paraformaldehyde solution were treated in a sequence using gradient dehydration, paraffin embedding, tissue slicing, hematoxylin-eosin staining, and microphotographic system observation for determining the diameter of the muscle fiber.

#### 2.2.5 Nutrient value evaluation

The amino acid score (AAS) is the ratio of the essential amino acid (EAA) content in the feed protein to the EAA content in the ideal pattern or reference protein. The ideal content of amino acids is the equilibrium pattern of the EAAs required by humans, which was revised in 1981 by the United Nations Food and Agriculture Organization (FAO)/World Health Organization (WHO), i.e., the amino acid content per gram protein [19]. The calculations are as follows:

-
\text{Amino}\hspace{.5em}\text{acid}\hspace{.5em}\text{score}\hspace{.25em}(\text{AAS})( \% )=\frac{\text{Amino acid content per gram protein in test proteinmg}}{\text{Amino acid content per gram protein in an ideal pattern or reference protein}}\times 100,]

-
\text{Amino acid content/}(\text{mg/g pro})\text{ }\frac{\text{Amino acid content }(\text{mg}/100\hspace{.1em}\text{g})}{\text{ Protein content }\left(\frac{\text{g}}{100\hspace{.1em}\text{g}}\right)\text{ }}.]

### 2.3 Data processing

The obtained data were analyzed using the SPSS 24.0 software. The data were analyzed by performing a one-way analysis of variance (ANOVA) and presented as the mean value ± standard deviation. All differences among and between groups were considered to be statistically significant at *P* < 0.05.

## 3 Results and discussion

### 3.1 Determining muscle nutrient composition

As shown in Table 1, protein content in the muscle tissue of QHMM (21.96 g/100 g) was higher than that of STH (20.70 g/100 g), but the difference was not significant (*P* > 0.05). However, the moisture contents of both breeds were higher than 75 g/100 g. However, the fat content of QHMM (1.8 g/100 g) was significantly lower (*P* < 0.05) than that of STH (3.1 g/100 g). A study [20] found that more than 70% of muscle tissue indicates a good muscle quality. To summarize, QHMM has high protein, low fat, and good muscle quality.

**Table 1:** The content of moisture, protein, and fat in muscle tissue (g/100 g)

Content	QHMM	STH
Moisture	75.23 ± 0.75	75.03 ± 1.00
Protein	21.96 ± 0.51	20.70 ± 1.75
Fat	1.80 ± 0.42*	3.10 ± 0.71

Note: Mean values within the same row with shoulder marker * indicates a significant difference (*P* < 0.05) between the data.

### 3.2 Analysis of amino acids in muscles and calculation of the AAS

The longissimus dorsi muscles of QHMM and STH were analyzed to determine the contents of amino acids. The results are presented in Table 2.

**Table 2:** The amino acid content in muscle tissue (g/100 g)

	Amino acid type	QHMM	STH
EAA	Threonine (Thr)	1.11 ± 0.02*	0.98 ± 0.06
	Valine (Val)	0.94 ± 0.05	0.87 ± 0.08
	Methionine (Met)	0.65 ± 0.08	0.59 ± 0.06
	Isoleucine (Ile)	0.96 ± 0.05	0.89 ± 0.13
	Leucine (Leu)	1.78 ± 0.06*	1.66 ± 0.18
	Phenylalanine (Phe)	0.95 ± 0.04	0.90 ± 0.07
	Lysine (Lys)	1.87 ± 0.14	1.74 ± 0.23
HEAA	Histidine (His)	0.99 ± 0.09*	0.89 ± 0.03
	Arginine (Arg)^△^	1.31 ± 0.12	1.27 ± 0.10
NEAA	Aspartate (Asp)^△^	2.04 ± 0.14*	1.78 ± 0.24
	Glutamate (Glu)^△^	3.38 ± 0.12*	2.91 ± 0.44
	Glycine (Gly)^△^	0.88 ± 0.04	0.81 ± 0.07
	Alanine (Ala)^△^	1.20 ± 0.05*	1.07 ± 0.07
	Serine (Ser)	0.87 ± 0.02*	0.76 ± 0.04
	Cystine (Cys)	0.31 ± 0.02	0.31 ± 0.05
	Tyrosine (Tyr)	0.96 ± 0.12*	0.85 ± 0.09
	Proline (Pro)	2.05 ± 0.30*	1.87 ± 0.29
	Hydroxyproline (Hpro)	0	0
TAA		22.26 ± 0.16*	20.16 ± 0.15
EAA		8.26 ± 0.10*	7.63 ± 0.11
HEAA		2.30 ± 0.10*	2.16 ± 0.11
NEAA		11.70 ± 0.04*	10.40 ± 0.08
FAA		8.82 ± 0.15*	7.84 ± 0.20
EAA/TAA(%)		37.00 ± 1.00	38.00 ± 1.00
EAA/NEAA(%)		71.00 ± 2.65	73.67 ± 3.05
FAA/TAA(%)		39.33 ± 1.15	39.00 ± 2.00

Note: The mean values in the same row that are marked with an asterisk indicate a significant difference (*P* < 0.05) between the values, and the values that are marked with a △ indicate flavor amino acids. EAA: essential amino acid, HEAA: half-essential amino acid, NEAA: non-essential amino acid, TAA: total amino acid, FAA: flavor amino acid.

As shown in Table 2, 17 amino acids were detected in both the sheep breeds with the same amino acid types and amino acid content sequences. These amino acids were classified into seven EAAs, including Thr, Val, Met, Ile, Leu, Phe, and Lys, two half-essential amino acids (HEAAs), including His and Arg, and eight non-essential amino acids (NEAAs), including Asp, Glu, Gly, Ala, Ser, Cys, Tyr, and Pro. Except for Cys, the amino acid content in the muscle tissue of QHMM was significantly higher than that of STH (*P* < 0.05) for the contents of Asp, Thr, Ser, Glu, Ala, Leu, Tyr, His, and Pro. Except for Cys, whose content in muscle tissue of STH was higher than that of QHMM, the content of other kinds of amino acids in muscle tissue of QHMM were higher than that of STH, and the content of Thr, Leu, His, Asp, Glu, Ala, Ser, Tyr, and Pro reached significant level (*p* < 0.05). In the muscle tissues of QHMM and STH, the five amino acids with the highest content in descending order were Glu, Pro, Asp, Lys, and Leu. Additionally, in the muscle tissues of QHMM and STH, the difference was significant (*P* < 0.05) for the contents of the total amino acid (TAA), EAA, HEAA, NEAA, and flavor amino acid (FAA). However, in the muscle tissues of these two sheep breeds, the difference was not significant (*P* > 0.05) for the values of EAA/TAA, EAA/NEAA, and FAA/TAA.

The protein content is an important index to measure nutritional muscle quality. The amino acid types and content in the muscle tissues can evaluate the protein nutrient value. The amino acid composition also plays an important role in meat flavor because they include many compounds that can produce precursors that affect flavor substances when heated. The essential amino acids in foods are particularly important because they maintain the vital activities of the human body [21]. In this study, the contents of Thr and Leu in the muscle tissue of QHMM were significantly higher than those of STH. These two amino acids belong to branched-chain amino acids (BCAAs). BCAA affects animal growth, production performance, and animal product quality by regulating glucose and lipid metabolism [22,23]. BCAA in the body is decomposed into acetoacetate, acetyl CoA, and succinyl COA through degradation and metabolism. It participates in the tricarboxylic acid cycle [24] through glycogenesis and ketogenesis, allows the mutual conversion of proteins, fats, and sugars, and increases the efficiency of biological functions. Arg and His are semi-essential amino acids in the human body. Arg can be converted into essential amino acids under certain conditions and is closely related to the juvenile growth of humans [14]. Here the contents of Arg and His in the muscle tissue of QHMM were higher than those of STH, and the difference was significant for His content. Thus, the content of HEAAs in the muscle tissue of QHMM was higher than that of STH. For proteins with high nutrient value, there should be a full range of EAAs and appropriate ratios between EAAs. In the ideal pattern of high-quality proteins proposed by FAO/WHO, the value of EAA/TAA is about 40%, whereas, the value of EAA/NEAA is higher than 60% [25]. In this study, the value of EAA/TAA in the muscle tissue was 37% for QHMM and 38% for STH, with no significant difference, and a value close to 40% for both breeds. On the other hand, the value of EAA/NEAA was 71% for QHMM and 73.67% for STH, with no significant difference statistically and a value higher than 60% for both breeds. Thus, the muscle tissues of these two sheep breeds were composed of high-quality proteins, and the amino acid content in the muscle tissue of QHMM was higher than that of STH. The deliciousness of animal proteins was closely related to the type and content of FAAs, and here there were five FAAs, including Glu, Arg, Gly, Ala, and Asp [25]. As shown in Table 2, five FAAs, including Glu, Arg, Gly, Ala, and Asp, were detected in QHMM and STH muscle tissues. The total content of FAA in the muscle tissue of QHMM was significantly higher (*P* < 0.05) than that of STH, but the value of FAA/TAA was 39.33% for QHMM and 39% for STH, and the difference was not significant (*P* > 0.05) for these two breeds. In the muscle tissues of these two breeds, the FAA content sequence in descending order was Glu, Asp, Arg, Ala, and Gly. The contents of Glu, Asp, and Ala in QHMM were significantly higher (*P* < 0.05) than those in STH. Glu and Asp are amino acids with umami taste features, while Gly and Ala are amino acids with sweet taste features [26]; these affect the flavor of sheep muscles [27]. Here we found that the muscle tissue of QHMM was rich in amino acids with umami taste features. The primary amino acids that affect the flavor of sheep muscles, including Glu and Asp, were among the five amino acids with the highest content in the muscle tissue of QHMM. Their contents in QHMM were significantly higher than those in STH. To summarize, the muscle tissue of QHMM was rich in delicious amino acids, and its umami taste features were better than those of STH.

Amino acid evaluation could be conducted based on the protein composition by calculating the AAS. In this study, the AAS calculation was performed through comparative analysis based on the equilibrium pattern of EAAs in the ideal protein proposed in 1981 by FAO/WHO. The results are shown in Table 3.

**Table 3:** The composition of EAAs in the muscle of QHMM and STH

Amino acid	FAO pattern (mg/g pro)	QHMM			STH		
		AA (mg/100 g dry sample)	AA (mg/g pro)	AAS (%)	AA (mg/100 g dry sample)	AA (mg/g pro)	AAS (%)
Threonine (Thr)	40.00	1108.67	50.49	126.21	976.67	47.18	117.96
Valine (Val)	50.00	941.00	42.85	85.70	873.67	42.21	84.41
Methionine + Cystine (Met + Cys)	35.00	959.67	43.70	124.86	901.67	43.56	124.45
Isoleucine (Ile)	40.00	960.00	43.72	109.29	894.67	43.22	108.05
Leucine (Leu)	70.00	1781.00	81.10	115.86	1661.33	80.26	114.65
Phenylalanine + Tyrosine (Phe + Tyr)	60.00	1909.00	86.93	144.88	1746.33	84.36	140.61
Lysine (Lys)	55.00	1872.67	85.28	155.05	1742.33	84.17	153.04

As shown in Table 3, the AAS values of threonine (Thr), methionine + cystine (Met + Cys), isoleucine (Ile), leucine (Leu), phenylalanine + tyrosine (Phe + Tyr), and lysine (Lys) in the muscle tissue of QHMM were higher than those of STH; the values of both sheep breeds were greater than those in the ideal pattern. The AAS value of valine (Val) in the muscle tissue of QHMM was greater than that of STH. Although the values of these two sheep breeds were lower than that in the ideal pattern, the contents of Val of both sheep breeds were close to that of the ideal pattern. The first limiting amino acid for both sheep breeds was Val. Therefore, if the value of AAS was considered to be the evaluation criterion, the contents of essential amino acids in the muscle tissues of these two sheep breeds were balanced and ideal, with the nutrient value of the muscle tissue of QHMM higher than that of STH.

### 3.3 Fatty acid content determination

The longissimus dorsi muscles of QHMM and STH were tested to determine the fatty acid content, and the results are shown in Table 4.

**Table 4:** The content of fatty acids in the muscle tissue

Fatty acid type		Abbreviation	QHMM	STH
MUFA (mg/100 g)	*Cis*-9 tetradecenoic acid	C14:1 n5	1.60 ± 0.37	1.64 ± 0.65
	*Cis*-10 pentadecenoic acid	C15:1 n5	3.05 ± 0.43	2.17 ± 0.14
	*Cis*-9 hexadecenoic acid	C16:1 n7	48.15 ± 2.56	51.06 ± 4.11
	*Cis*-9 octadecenoic acid	C18:1 n9	869.15 ± 49.20	862.75 ± 15.50
	*Cis*-11 eicosenoic acid	C21:1	1.59 ± 0.67	1.31 ± 0.05
	*Cis*-13 docosenoic acid	C22:1 n9	0.59 ± 0.13*	0.15 ± 0.06
PUFA (mg/100 g)	*Cis*-9, 12 octadecadienoic acid	C18:2 n6	76.44 ± 13.70*	68.06 ± 3.56
	*Cis*-6, 9, 12 octadecatrienoic acid	C18:3 n6	0.57 ± 0.13*	0.24 ± 0.04
	*Cis*-9, 12, 15 octadecatrienoic acid	C18:3 n3	6.42 ± 0.32	6.74 ± 0.86
	*Cis*-11, 14 eicosadienoic acid	C20:2	0.20 ± 0.03	ND
CDFC	*Cis*-8, 11, 14 eicosatrienoic acid	C20:3 n6	0.69 ± 0.07	0.42 ± 0.03
	*Cis*-5, 8, 11, 14 eicosatetraenoic acid	C20:4 n6	10.78 ± 1.80	7.10 ± 1.32
	EPA/*cis*-5, 8, 11, 14, 17 eicosapentaenoic acid	C22:5 n3	1.57 ± 0.05	1.05 ± 0.03
	DHA/*cis*-4, 7, 10, 13, 16, 19 docosahexaenoic acid	C22:6 n6	1.18 ± 0.13*	0.78 ± 0.08
SFA (mg/100 g)	Capric acid	C10:0	0.93 ± 0.17*	0.39 ± 0.06
	Undecanoic acid	C11:0	ND	0.01 ± 0.001
	Dodecanoic acid	C12:0	1.09 ± 0.11	2.63 ± 0.27 *
	Tetradecanoic acid	C14:0	52.05 ± 6.28	56.31 ± 7.41
	Pentadecanoic acid	C15:0	9.16 ± 0.43	9.69 ± 1.67
	Hexadecanoic acid	C16:0	594.80 ± 45.81	598.11 ± 40.56
	Heptadecanoic acid	C17:0	26.29 ± 1.46	27.60 ± 3.04
	Octadecanoic acid	C18:0	431.29 ± 28.11	460.00 ± 36.54
	Eicosanoic acid	C20:0	1.93 ± 0.56	2.06 ± 0.22
	Tricosanoic acid	C23:0	0.003 ± 0.0005	ND
MUFA (g/100 g)			0.97 ± 0.15	0.97 ± 0.16
PUFA (g/100 g)			0.10 ± 0.04	0.08 ± 0.01
UFA (g/100 g)			1.02 ± 0.16	1.00 ± 0.17
SFA (g/100 g)			1.07 ± 0.16	1.11 ± 0.22
UFA/SFA			0.95 ± 0.06	0.90 ± 0.03
PUFA/SFA			0.09 ± 0.01	0.07 ± 0.01

Note: Mean values within the same row with shoulder marker * indicate a significant difference (*P* < 0.05) between the data. MUFA: monounsaturated fatty acid, PUFA: polyunsaturated fatty acid, UFA: unsaturated fatty acid, SFA: saturated fatty acid.

As shown in Table 4, 23 fatty acids were found in the longissimus dorsi muscle of QHMM, including 6 monounsaturated fatty acids (MUFAs), 8 polyunsaturated fatty acids (PUFAs), and 9 saturated fatty acids (SFAs). Moreover, 22 fatty acids were detected in the longissimus dorsi muscle of STH, including 6 MUFAs, 7 PUFAs, and 9 SFAs. Among MUFAs, the content of C22:1 n9 in QHMM was significantly higher (*P* < 0.05) than that in STH. Among PUFAs, the contents of C18:2 n6, C18:3 n6, and C22:6 were considerably higher in QHMM (*P* < 0.05) than those in STH. Among SFAs, the content of C10:0 in QHMM was significantly higher (*P* < 0.05) than that in STH. Additionally, C11:0 was not found in QHMM, while C20:2 and C23:0 were not detected in STH.

The fatty acids in animal muscle tissues are divided into the following three categories: MUFA, PUFA, and SFA. Public health institutions pay close attention to the intake of dietary fatty acids. It is recommended to consume a balanced proportion of SFA, MUFA, and PUFA to maintain an appropriate diet and healthy lifestyle. This prompted us to study the fatty acid composition of QHMM and evaluate some parameters that directly affect human health. This study showed that the fatty acid composition in the muscle tissue was consistent for QHMM and STH and followed the order SFA > MUFA > PUFA. Thus, among fatty acids, the content of SFA was the highest, followed by MUFA, and the content of PUFA was the lowest. This was consistent with the fact that the proportion of SFAs in animal fatty acids is higher than that of vegetable oils and fats. The fatty acid content is another important index for determining the nutritive quality of the muscle; different fatty acids have different biological functions. MUFA reduces blood sugar and cholesterol content and adjusts the range of blood fat [28]. PUFA reduces cholesterol content, controls genetic expressions, and combats inflammations and tumors [29]. Some studies have shown that cardiovascular damage might be caused in humans by excessive intake of SFA. However, SFA can perform important biological functions and prevent and control diseases such as alcoholic liver disease [30]. This study showed that the contents of C18:2 n6, C18:3 n6, C22:6 n6, C22:1 n9, and C10:0 in the longissimus dorsi muscle of QHMM were significantly higher (*P* < 0.05) than those in the muscles of STH. Of these, the contents of C18:2 n6 and C10:0 are important factors that affect the flavor and umami odor features of sheep muscles [31,32]. The UFA/SFA of the longissimus dorsi muscle was 0.95 for QHMM and 0.90 for STH; the results were similar to those reported in a previous study [33]. This ratio affects the flavor and taste of muscle tissues, and the higher the ratio, the better the flavor [34]. Thus, we found that the flavor profile of QHMM was better than that of STH. Among the SFAs identified from the samples, C16:0, C18:0, C14:0, C17:0 and C15:0 (the top five SFAs with the largest proportion) were also similar to those found in previous studies [33,35,36]. Moreover, the PUFA:SFA ratio of the longissimus dorsi muscle was 0.09 for QHMM and 0.07 for STH. The common PUFA:SFA ratio was about 0.1 for livestock and poultry meat products. Although these PUFA:SFA values were somewhat lower than the FAO/WHO-recommended PUFA:SFA ratio of higher than 0.4 [37], this situation was consistent with the characteristics of livestock and poultry meat products. Previous studies have demonstrated a negative correlation between the PUFA: SFA ratio and cardiovascular diseases, suggesting that a balanced diet with PUFA instead of SFA can reduce the risk of these diseases [38]. To summarize, the types of fatty acids in the muscle tissues of QHMM were rich and had high nutrient values, and the taste and flavor of the muscle tissue of QHMH were better than those of STH.

### 3.4 Muscle fiber diameter determination

**Figure 2:**
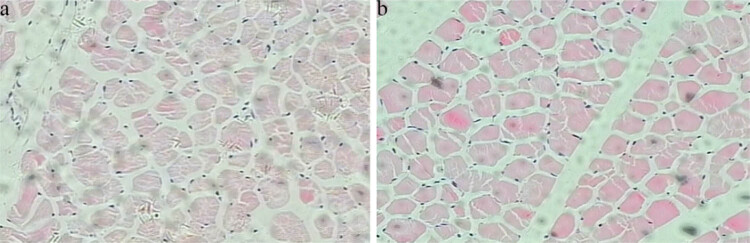
The longissimus muscle fiber of QHMM and STH with a magnification factor of 20 × 10: (a) QHMM and (b) STH.

**Figure 3:**
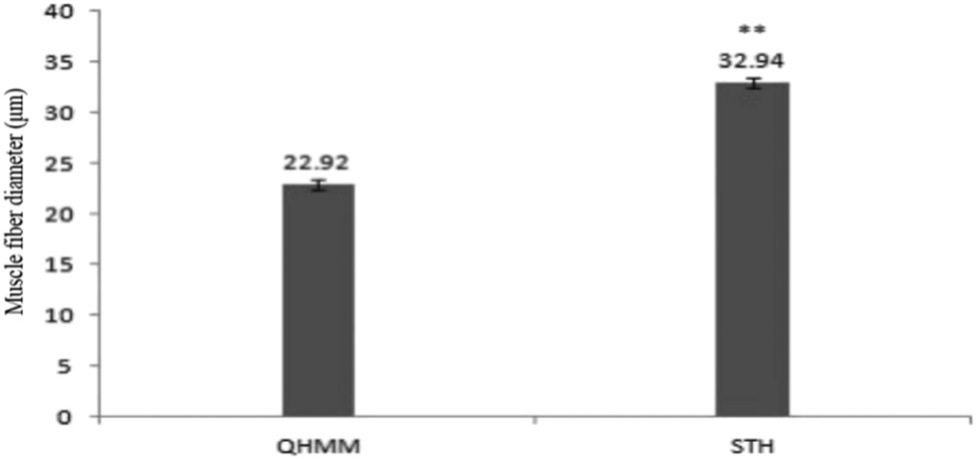
The longissimus muscle fiber of Qianhua Mutton Merino and Small-tail Han sheep. Note: The shoulder marker ** indicates a significant difference (*P* < 0.01).

The muscle tissues of QHMM and STH were tested to determine the diameter of the muscle fiber, and the results are shown in Figures 2 and 3.

As shown in Figure 3, the muscle fiber diameter of the longissimus dorsi muscle of QHMM was significantly lower (*P* < 0.01) than that of STH.

Muscle fibers are the fundamental components of animal skeletal muscles, and therefore, the muscle fiber diameter is closely related to muscle quality [39]. Some studies showed that the muscle fiber diameter was directly correlated with muscle tenderness, and the smaller the muscle fiber diameter, the better the muscle tenderness. In contrast, a larger diameter of muscle fibers was associated with poorer muscle tenderness [40]. Additionally, muscle fiber diameter is also affected by the animal breed [40,41]. In this study, we tested the longissimus dorsi muscles of QHMM and STH to determine the muscle fiber diameter and found that the diameter of QHMM was more significant than that of STH. Thus, the muscle tenderness and quality of QHMM were better than that of STH.

## 4 Conclusion

In conclusion, specific differences between the two sheep breeds concerning muscle quality were observed in this study. Qianhua Mutton Merino is a mutton sheep breed that produces high-quality mutton with high nutrient value, which is delicious and has certain healthcare benefits. Compared to the existing local sheep breed of STH, Qianhua Mutton Merino had a better meat quality and higher and more balanced nutrient values; thus, they can be used to produce high-quality meat products suitable for humans. In this study, research on the muscle quality of the new sheep breed of Qianhua Mutton Merino was conducted. Here we provided new information on the muscle nutrient composition of this sheep breed. A comparative study on muscle quality between the new sheep breed and the existing local sheep breed of STH provided a theoretical foundation for further detailed studies on Qianhua Mutton Merino. The study might act as a reference and guide the cultivation and breeding of Qianhua Mutton Merino.
